# Aspirin attenuates the detrimental effects of TNF-α on BMMSC stemness by modulating the YAP-SMAD7 axis

**DOI:** 10.1186/s10020-024-00890-z

**Published:** 2024-08-16

**Authors:** Xudong Wang, Yong Liu, Shiyong Zhang, Linli Zheng, Yunze Kang, Puyi Sheng, Ziji Zhang

**Affiliations:** 1 Department of Orthopedics, the First Affiliated Hospital of Sun Yat-sen University, #58 Zhongshan Road II, Guangzhou, Guangdong 510080 China; 2 Guangdong Provincial Key Laboratory of Orthopedics and Traumatology, the First Affiliated Hospital of Sun Yat-sen University, Guangzhou, Guangdong China

**Keywords:** BMMSCs, Aspirin, TNF-α, Stemness, YAP, SMAD7

## Abstract

**Background:**

Bone marrow mesenchymal stem cells (BMMSCs) are commonly used for cell transplantation to treat refractory diseases. However, the presence of inflammatory factors, such as tumour necrosis factor-alpha (TNF-α), at the transplantation site severely compromises the stemness of BMMSCs, thereby reducing the therapeutic effect of cell transplantation. Aspirin (AS) is a drug that has been in use for over a century and has a wide range of effects, including the regulation of cell proliferation, multidirectional differentiation, and immunomodulatory properties of stem cells. However, it is still unclear whether AS can delay the damaging effects of TNF-α on BMMSC stemness.

**Methods:**

This study investigated the effects of AS and TNF-α on BMMSC stemness and the molecular mechanisms using colony formation assay, western blot, qRT-PCR, and overexpression or knockdown of YAP and SMAD7.

**Results:**

The results demonstrated that TNF-α inhibited cell proliferation, the expression of stemness, osteogenic and chondrogenic differentiation markers of BMMSCs. Treatment with AS was shown to mitigate the TNF-α-induced damage to BMMSC stemness. Mechanistic studies revealed that AS may reverse the damage caused by TNF-α on BMMSC stemness by upregulating YAP and inhibiting the expression of SMAD7.

**Conclusion:**

AS can attenuate the damaging effects of TNF-α on BMMSC stemness by regulating the YAP-SMAD7 axis. These findings are expected to promote the application of AS to improve the efficacy of stem cell therapy.

**Supplementary Information:**

The online version contains supplementary material available at 10.1186/s10020-024-00890-z.

## Introduction

Bone marrow mesenchymal stem cells (BMMSCs) are commonly used for cell transplantation due to their abundant cell source, low immunogenicity(Ankrum et al. 2014), genetic stability, and ethical acceptability(Naji et al. 2019). BMMSCs can differentiate into various tissues, such as bone, cartilage, adipose, tendon, and so on. Therefore, they have been widely used to treat refractory diseases, including ischaemic heart disease(Ward et al. 2018), chronic lung transplantation syndrome(Erasmus et al. 2022), spinal cord injury(Li et al. 2023), osteoarthritis(Yu et al. 2022), osteoporosis(Liu et al. 2021), and other conditions. However, the clinical effectiveness of BMMSCs is restricted by the presence of inflammatory factors, such as tumour necrosis factor-alpha (TNF-α), at the site of cell transplantation(Wang et al. 2019a, b). Therefore, finding ways to delay or even reverse the desiccating damage caused by TNF-α on BMMSCs is important to improve the efficacy of cell transplantation.

Aspirin (AS) is a widely used antipyretic and analgesic with various effects. Studies have demonstrated that AS can regulate stem cell functions, such as regulating stem cell proliferation, multidirectional differentiation, and immune properties. For example, Hao et al. (Hao et al. 2018). reported that AS inhibited the growth of BMMSCs and promoted their differentiation into cardiomyocytes. Similarly, Cao et al. (Cao et al. 2015). found that AS promoted the osteogenic differentiation of BMMSCs, leading to the repair of bone defects in miniature pigs. In contrast, Zhan et al. (Zhan et al. 2018). reported that AS inhibited the osteogenic differentiation of BMMSCs. Wang et al. (Wang et al. 2023). found that AS could delay the inhibitory effect of TNF-α on the chondrogenic differentiation of BMMSCs. Additionally, AS has been shown to improve the immunomodulatory properties of BMMSCs by modulating the 15d-PGJ2/PPARγ/TGF-β1 pathway(Tang et al. 2014). However, no studies have been conducted to determine whether AS can delay TNF-α-induced desiccation of BMMSC stemness.

In this study, we aim to explore the roles and molecular mechanisms of AS and TNF-α on BMMSC stemness, which may advance the application of AS to improve the efficacy of stem cell therapy.

## Materials and methods

### Isolation and culture of BMMSCs

This study has been approved by the the Research Ethics Committee of the First Affiliated Hospital of Sun Yat-sen University. Written informed consent was obtained from each volunteer. Referring to previously published literature(Wang et al. 2019a, b, 2021a, b, 2023; Qiu et al. 2019; Gao et al. 2018; Wu et al. 2018), the BMMSCs used in this study were extracted from healthy volunteers and isolated using density gradient centrifugation (500 g for 20 min at 37℃). They were then cultured in a low-sugar DMEM medium supplemented with 10% fetal bovine serum (FBS), penicillin(100 U/ml), and streptomycin(100 U/ml). The medium was changed every 3 days, and the cells were passaged when the cell density reached 80–90%. The 3rd to 6th generations of BMMSCs were used for subsequent experiments.

### Antibodies and reagents

TNF-α and AS were purchased from MedChemExpress (New Jersey, USA). Antibodies against C-MYC(ab32072), NANOG(ab109250), SOX2(ab92494), OCN(ab133612), OPN(ab214050), RUNX2(ab192256), COL2A1(ab34712), ACAN(ab3778), SOX9(ab185966), YAP(ab52771), SMAD7(ab216428) and GAPDH(ab9485) were purchased from Abcam (Cambridge, UK). Goat anti-mouse IgG H&L (HRP)(#14709) and Goat anti-rabbit IgG H&L (HRP)(#35401) were purchased from Cell Signaling Technology (Boston, USA).

### Colony formation experiment

BMMSCs in the logarithmic growth phase from different treatment groups were digested and resuspended to determine the cell count. The cells were then plated in 6-well plates at a density of 1500 cells per well, with the medium being changed every 3 days. The formation of cell colonies was observed under a microscope after approximately 14 days of culture. The cells were fixed with 4% paraformaldehyde (Phygene, Fujian, China) for 30 min. Subsequently, they were stained with a 1% crystal violet solution (Servicebio, Wuhan, China) for 5 min, washed 3 times with deionized water, and then scanned to determine the number of cell colonies.

### Induction of BMMSCs osteogenic differentiation

BMMSCs in the logarithmic growth phase were digested and resuspended. The cells were then inoculated into 6-well plates coated with gelatin. After 24 h of cell attachment, the medium was replaced with BMMSCs Osteogenic Induction and Differentiation Medium (Phygene, China) for osteogenic differentiation induction for 14 days. The medium was changed every 3 days.

### Induction of BMMSCs chondrogenic differentiation

BMMSCs in the logarithmic growth phase were disrupted and resuspended. The cells were then inoculated into gelatin-coated 6-well plates and left to adhere for 24 h. Mesenchymal stem cell chondrogenic differentiation medium (ScienCell, USA) was then added to induce chondrogenic differentiation for 14 days, with medium changes every 3 days.

### Western blot experiment

BMMSCs with different treatments were lysed using RIPA protein lysate (Beyotime, USA). The lysates were vigorously shaken for 30 s and then placed on ice for 10 min, and the above processes were repeated three times. The lysates were then centrifuged at high speed, and the supernatants were extracted. The protein concentrations were measured using a BCA Protein Concentration Determination Kit (Beyotime, USA). The protein concentrations were equalized using RIPA lysate and 5× loading buffer solution. The proteins were then separated using electrophoresis on a gel (EpiZyme, China) and transferred to a polyvinylidene fluoride (PVDF) membrane. Subsequently, the membranes were immersed in a TBST solution containing 5% milk at room temperature for 1 h. After that, they were incubated overnight at 4 °C with different antibodies (dilution 1:1000). The following day, the membranes were incubated with the secondary antibody (dilution 1:2000) at room temperature for 1 h. The protein bands were then visualized using an HRP Chemiluminescent Western Blot Detection Kit (Merck Millipore, USA).

### qRT-PCR experiment

RNA was extracted from BMMSCs of different treatment groups using an RNA extraction kit (GIBCO, USA). The RNA concentrations were detected using a spectrophotometer (Thermo Fisher Scientific, USA) and then converted to cDNA using the Evo M-MLVRT Kit (#AG11706; Accurate Biotechnology, Hunan, China). The cDNAs were amplified and quantified using the SYBR Green Pro Taq HS Premix II Kit (#AG11702; Accurate Biotechnology, Hunan, China). The primer sequences of the genes were listed in Table 1.

**Table 1:** Sequences of primers used for quantitative PCR.

Gene	Genebank ID	Primer sequence (5’-3’)
*GAPDH*	2597	Forward: GGAGCGAGATCCCTCCAAAAT; Reverse: GGCTGTTGTCATACTTCTCATGG;
*C-MYC*	4609	Forward: GGCTCCTGGCAAAAGGTCA; Reverse: CTGCGTAGTTGTGCTGATGT;
*NANOG*	79,923	Forward: TTTGTGGGCCTGAAGAAAACT; Reverse: AGGGCTGTCCTGAATAAGCAG;
*SOX2*	6657	Forward: GCCGAGTGGAAACTTTTGTCG; Reverse: GGCAGCGTGTACTTATCCTTCT;
*OCN*	632	Forward: CACTCCTCGCCCTATTGGC; Reverse: CCCTCCTGCTTGGACACAAAG;
*OPN*	6696	Forward: CTCCATTGACTCGAACGACTC; Reverse: CAGGTCTGCGAAACTTCTTAGAT;
*RUNX2*	860	Forward: TGGTTACTGTCATGGCGGGTA; Reverse: TCTCAGATCGTTGAACCTTGCTA;
*COL2A1*	1280	Forward: GCTGCGGATGCTCTCAATCT; Reverse: TGGACGATCAGGCGAAACC;
*ACAN*	176	Forward: ACTCTGGGTTTTCGTGACTCT; Reverse: ACACTCAGCGAGTTGTCATGG;
*SOX9*	6662	Forward: AGCGAACGCACATCAAGAC; Reverse: CTGTAGGCGATCTGTTGGGG;
*YAP*	10,413	Forward: TAGCCCTGCGTAGCCAGTTA; Reverse: TCATGCTTAGTCCACTGTCTGT;
*SMAD7*	4092	Forward: TTCCTCCGCTGAAACAGGG; Reverse: CCTCCCAGTATGCCACCAC;

### The construction of plasmids and lentivirus

Plasmids were obtained from Addgene and transfected using Lipofectamine 3000 reagent according to the manufacturer’s instructions. Lentiviral transfer plasmids, pSin-YAP-FLAG(#66853) and pSin-SMAD7-FLAG(#141518), were created by cloning the human YAP and SMAD7 coding sequences into the plasmid pSin-puro. A control vector plasmid was also constructed. To silence the expression of YAP and SMAD7, the shRNA sequence was cloned into the lentiviral transfer plasmids pSin-YAP shRNA(sequence: GCCACCAAGCTAGATAAAGAA) and pSin-SMAD7 shRNA(sequence: GCAGACAGACACTGGATTAAA). A control scramble-shRNA(sequence: GCGCGCTTTGTAGGATTCG) was also constructed. Lentiviral infection was performed according to the procedure outlined in a previously published article(Lian et al. 2019).

### Statistical analysis

The quantitative data were analyzed using SPSS 23.0 software. Student’s t-test was used to determine differences between two groups, while analysis of variance was used to determine differences between more than two groups. A p-value of less than 0.05 was considered statistically significant. Notations used include: “*” for *P* < 0.05, “**” for *P* < 0.01, and “***” for *P* < 0.001.

## Results

### AS reversed the damaging effect of TNF-α on BMMSC stemness

**Fig. 1:**
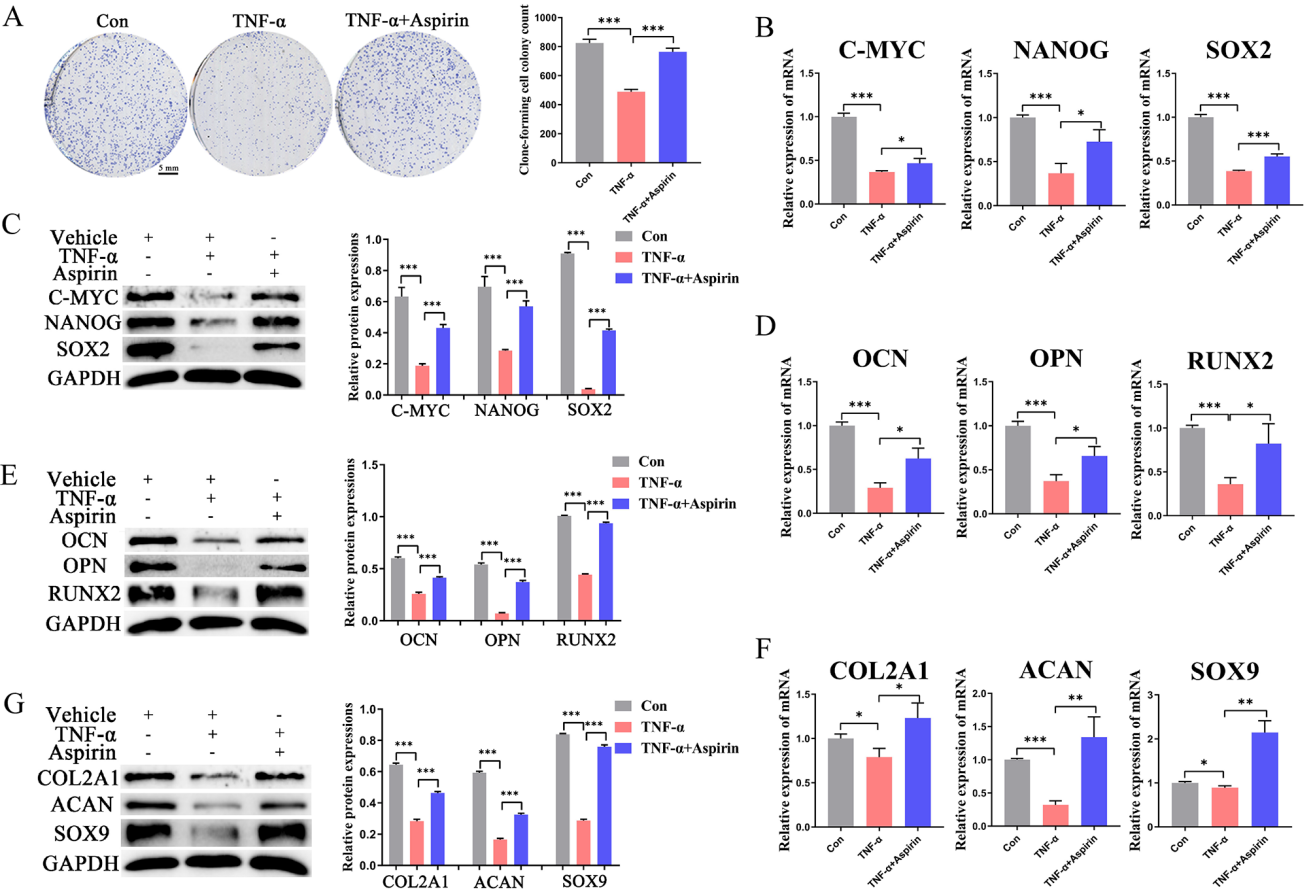
Effect of TNF-α and aspirin on the stemness of BMMSCs.(**A**) Representative pictures and quantitative results of colony formation of BMMSCs with different treatments; (**B**, **C**) The expression of stemness markers (C-MYC, NANOG, SOX2) of BMMSCs with different treatments were analyzed using qRT-PCR and western blot assays. (**D**, **E**) The expression of osteogenic differentiation markers (OCN, OPN, RUNX2) of BMMSCs in different groups were analyzed using qRT-PCR and western blot experiments. (**F**, **G**) The expression of chondrogenic differentiation markers (COL2A1, ACAN, SOX9) in BMMSCs from different groups were analyzed using qRT-PCR and western blot assays. Data in A-F are given as the mean ± SD of three independent experiments. *P < 0.05, **P < 0.01, ***P < 0.001. Scale bars: 5 mm

Human BMMSCs were pretreated with AS (100 µM) or TNF-α (10 ng/ml) for 5 days. The cells were then induced to undergo osteogenic and chondrogenic differentiation for 14 days. The expression of proliferation and stemness markers, as well as osteogenic and chondrogenic differentiation markers, were detected in BMMSCs after 14 days. The results showed that TNF-α impaired the self-renewal and multidirectional differentiation ability (stemness) of BMMSCs, as evidenced by the inhibition of BMMSCs proliferation (Fig. 1A), the downregulation of stemness markers (C-MYC, NANOG, and SOX2) (Fig. 1B, C), osteogenic differentiation markers (OCN, OPN, and RUNX2) (Fig. 1D, E), and chondrogenic differentiation markers (COL2A1, ACAN, and SOX9) (Fig. 1F, G). However, pre-treatment with AS attenuated these inhibitory effects, suggesting that AS delayed TNF-α-induced damage to the stemness of BMMSCs.

### AS attenuated TNF-α damage to BMMSC stemness by up-regulating YAP

**Fig. 2:**
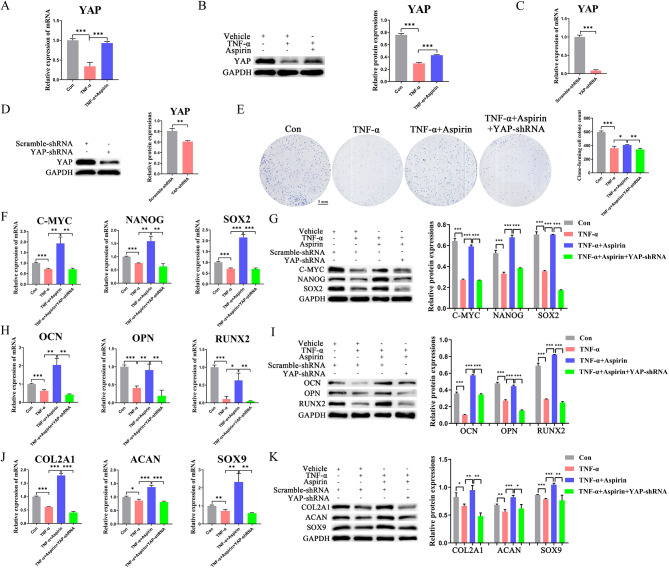
Inhibition of YAP reverses the protective effect of AS on BMMSC stemness (**A**, **B**) YAP in different treatment groups was determined using qRT-PCR and western blot assays with or without the AS and TNF-α treatments; (**C**, **D**) Detection of YAP knockdown efficiency using qRT-PCR and western blot assays; (**E**) Representative pictures and quantitative results of colony formation of BMMSCs with different treatments; (**F**, **G**) The expression of stemness markers (C-MYC, NANOG, SOX2) of BMMSCs in different groups were analyzed using qRT-PCR and western blot experiments. (**H**, **I**) The expression of osteogenic differentiation markers (OCN, OPN, RUNX2) of BMMSCs in different groups were analyzed using qRT-PCR and western blot experiments. (**J**, **K**) The expression of chondrogenic differentiation markers (COL2A1, ACAN, SOX9) in BMMSCs with different treatments were analyzed using qRT-PCR and western blot assays. Data in A-K are given as the mean ± SD of three independent experiments. *P < 0.05, **P < 0.01, ***P < 0.001. Scale bars: 5 mm

We further investigated the molecular mechanisms by which AS reversed the TNF-α-induced BMMSC stemness damage and found that TNF-α suppressed the expression of YAP, whereas AS treatment up-regulated the expression of YAP (Fig. 2A, B). To further validate the role of YAP, we knocked down the YAP of BMMSCs (Fig. 2C, D). We found that knockdown of YAP partially reversed the promotion of AS on cell proliferation (Fig. 2E), the expression of stemness markers (Fig. 2F, G), osteogenic differentiation markers (Fig. 2H, I) and chondrogenic differentiation markers (Fig. 2J, K). This strongly suggests that YAP is involved in mediating the reversal effect of AS on BMMSC stemness.

### Overexpression of YAP reversed the damaging effect of TNF-α on BMMSC stemness

**Fig. 3:**
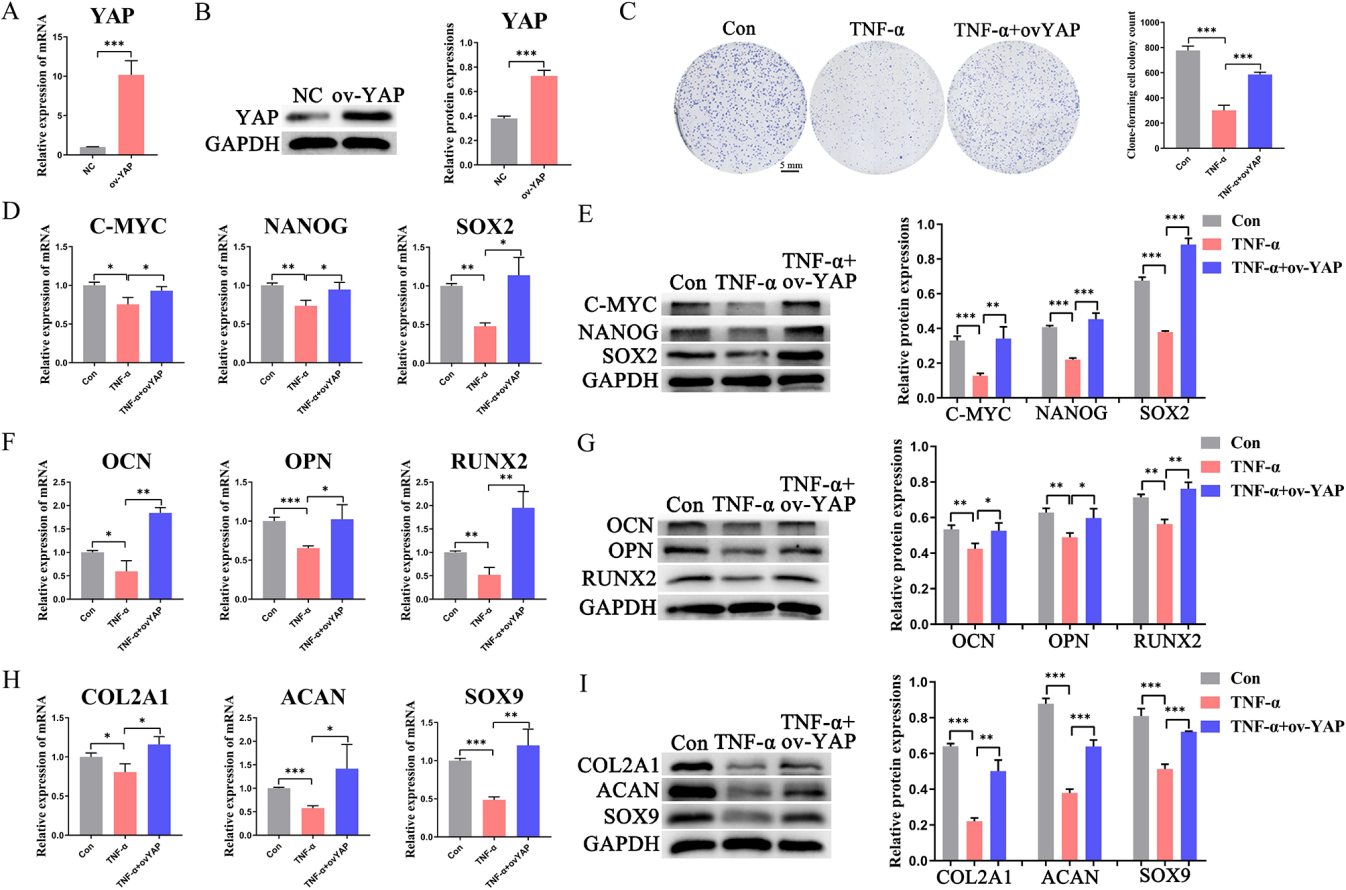
Overexpression of YAP attenuates the damaging effects of TNF-α on BMMSC stemness (**A**, **B**) Detection of YAP overexpression efficiency using qRT-PCR and western blot assays; (**C**) Representative pictures and quantitative results of colony formation of BMMSCs with different treatments; (**D**, **E**) The expression of stemness markers (C-MYC, NANOG, SOX2) of BMMSCs in different groups were analyzed using qRT-PCR and western blot experiments. (**F**, **G**) The expression of osteogenic differentiation markers (OCN, OPN, RUNX2) of BMMSCs in different groups were analyzed using qRT-PCR and western blot experiments. (**H**, **I**) The expression of chondrogenic differentiation markers (COL2A1, ACAN, SOX9) in BMMSCs from different groups were analyzed using qRT-PCR and western blot assays. Data in A-I are given as the mean ± SD of three independent experiments. *P < 0.05, **P < 0.01, ***P < 0.001. Scale bars: 5 mm

To further verify that AS can reverse the stemness-damaging effect of TNF-α on BMMSCs by upregulating YAP, we overexpressed YAP (Fig. 3A, B) and found that overexpression of YAP partially reversed the effects of TNF-α on cell proliferation (Fig. 3C), stemness markers (Fig. 3D, E), osteogenic differentiation markers (Fig. 3F, G) and chondrogenic differentiation markers (Fig. 3H, I). These findings indicated that YAP regulated the maintenance of stemness in BMMSCs, similar to AS treatment.

### YAP may regulate BMMSC stemness through binding to SMAD7

**Fig. 4:**
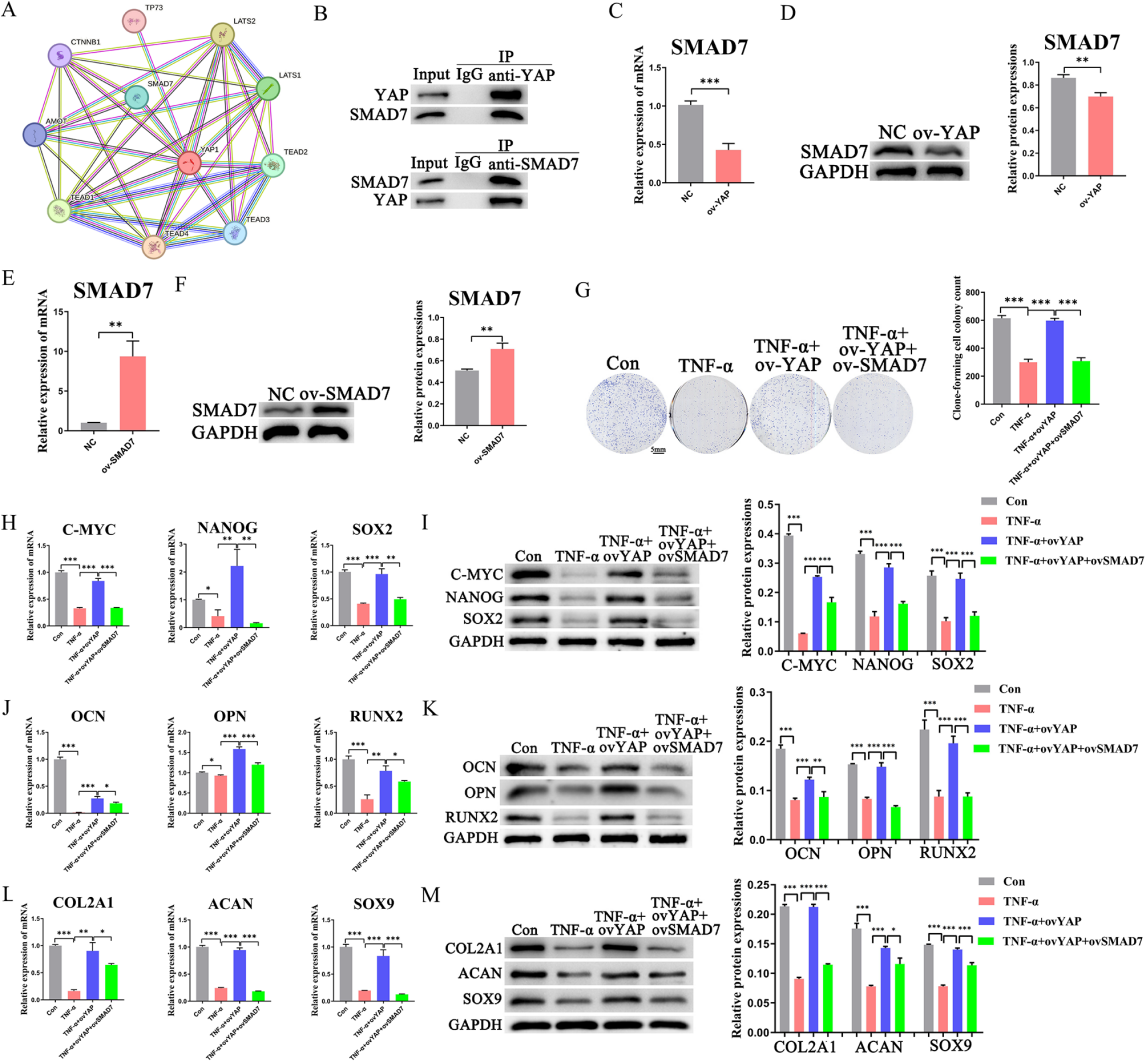
Overexpression of SMAD7 attenuates the protective effects of YAP on the stemness of BMMSCs (**A**) The prediction of proteins that may bind to YAP was carried out using the STRING website; (**B**) Co-IP experiments were conducted to detect YAP binding to SMAD7; (**C**, **D**) qRT-PCR and western blot experiments were performed to detect SMAD7 expression in BMMSCs after overexpression of YAP; (**E**, **F**)Detection of SMAD7 overexpression efficiency using qRT-PCR and western blot assays G Representative pictures of colony formation of BMMSCs with different treatments; (**H**, **I**) The expression of stemness markers (C-MYC, NANOG, SOX2) of BMMSCs in different groups were analyzed using qRT-PCR and western blot experiments. (**J**, **K**) The expression of osteogenic differentiation markers (OCN, OPN, RUNX2) of BMMSCs in different groups were analyzed using qRT-PCR and western blot experiments. (**L**, **M**) The expression of chondrogenic differentiation markers (COL2A1, ACAN, SOX9) in BMMSCs with different treatments were analyzed using qRT-PCR and western blot assays. Data in C-M are given as the mean ± SD of three independent experiments. *P < 0.05, **P < 0.01, ***P < 0.001. Scale bars: 5 mm

To investigate the downstream mechanism of YAP, we used the STRING website(https://cn.string-db.org/) to predict potential protein interactions. Our analysis revealed that YAP may bind to SMAD7, TP73, CTNNB1, LATS1, LATS2, TEAD1, and TEAD2 (Fig. 4A). As the BMP/SMAD pathway regulates stem cell function, we chose to validate the SMAD7 due to its significant role as a negative regulator of this pathway. The result revealed a protein interaction between YAP and SMAD7 through immunoprecipitation (Fig. 4B) and ChIP-qPCR (Supplementary Fig. 1). Moreover, the overexpression of YAP suppressed the expression of SMAD7 (Fig. 4C, D), indicating that YAP may regulate stem cell function by mediating the expression of SMAD7. To further confirm that YAP exerts its mediating effect through the downregulation of SMAD7, we overexpressed SMAD7 and verified its efficiency (Fig. 4E, F). We subsequently co-overexpressed YAP and SMAD7 and found that the overexpression of SMAD7 delayed the effect of YAP overexpression on cell proliferation (Fig. 4G), stemness markers (Fig. 4H, I), osteogenic differentiation markers (Fig. 4J, K), and chondrogenic differentiation markers (Fig. 4L, M). Taken together, YAP may regulate the stemness-maintaining effect of AS on BMMSCs by binding to SMAD7 and downregulating its expression.

### Knockdown of SMAD7 reverses the stemness-damaging effect of TNF-α on BMMSCs

**Fig. 5:**
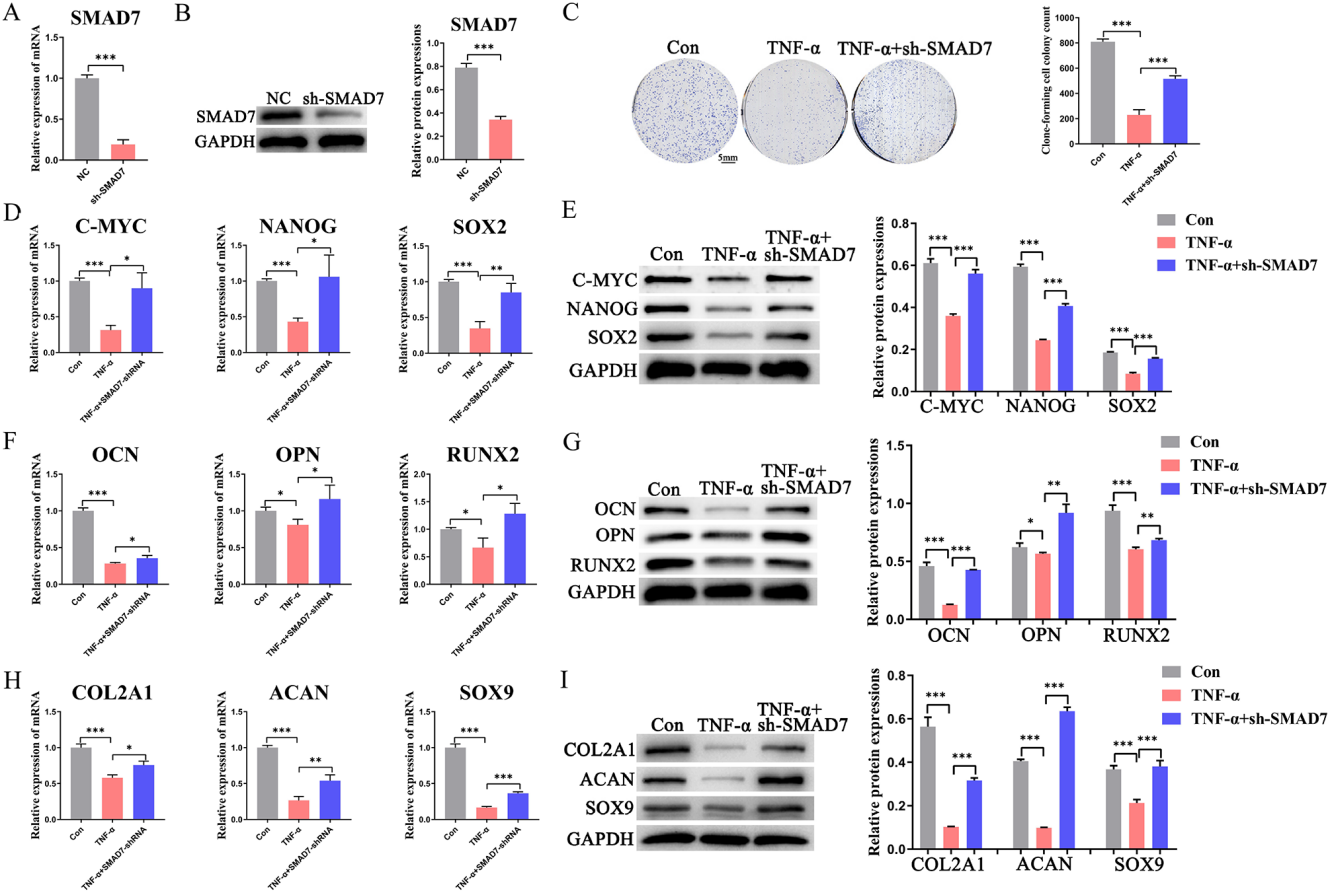
Inhibition of SMAD7 reverses the damaging effects of TNF-α on BMMSC stemness (**A**, **B**) Detection of SMAD7 knockdown efficiency using qRT-PCR and western blot assays; (**C**)Representative pictures and quantitative results of colony formation of BMMSCs with different treatments; (**D**, **E**) The expression of stemness markers (C-MYC, NANOG, SOX2) of BMMSCs in different groups were analyzed using qRT-PCR and western blot experiments. (**F**, **G**) The expression of osteogenic differentiation markers (OCN, OPN, RUNX2) of BMMSCs in different groups were analyzed using qRT-PCR and western blot experiments. (**H**, **I**) The expression of chondrogenic differentiation markers (COL2A1, ACAN, SOX9) in BMMSCs from different groups were analyzed using qRT-PCR and western blot assays. Data in A-I are given as the mean ± SD of three independent experiments. *P < 0.05, **P < 0.01, ***P < 0.001. Scale bars: 5 mm

To further validate that YAP can reverse the stemness-damaging effect of TNF-α on BMMSCs by downregulating SMAD7, we knocked down SMAD7 (Fig. 5A, B) and found that SMAD7 knockdown partially reversed the effects of TNF-α on cell proliferation (Fig. 5C), stemness markers (Fig. 5D, E), osteogenic differentiation markers (Fig. 5F, G) and chondrogenic differentiation markers (Fig. 5H, I). The results indicate that knockdown of SMAD7 can produce effects similar to AS treatment or overexpression of YAP, suggesting a mediating role on BMMSC stemness for SMAD7.

## Discussion

Given the paucity of research exploring the impact of AS on the maintenance of stem cell stemness, this study makes a novel contribution to this field by demonstrating that AS mitigates damage of the inflammatory factor TNF-α on the stemness of BMMSCs. Mechanistically, AS may exert this reversal effect by upregulating the expression of YAP and downregulating the expression of SMAD7. The innovations of this study include the following two main points: (1) The first to propose that AS can delay inflammation-induced stemness damage of BMMSCs; (2) The first to propose the important regulatory role of the YAP-SAMD7 signalling axis in maintaining the stemness of BMMSCs. The results of this study are expected to promote the application of AS in maintaining the stemness of BMMSCs, thereby improving the efficacy of cell transplantation and providing new hope for the treatment of many difficult and complicated diseases.

Stem cell stemness refers to the ability of stem cells to maintain self-renewal and multidirectional differentiation. Maintaining stem cell stemness is essential for improving the efficacy of cell transplantation(Teng et al. 2018). However, with the increase in stem cell passaging in vitro and the detrimental effects of inflammatory factors such as TNF-α on stem cell stemness, the therapeutic efficacy of stem cells is severely compromised. In this study, we treated BMMSCs with TNF-α at a concentration of 10 ng/ml and found that TNF-α inhibited cell proliferation and the ability of BMMSCs to differentiate in multiple directions, suggesting that the stemness of BMMSCs was impaired. Previous studies have also shown that TNF-α impairs the stemness of stem cells by down-regulating YAP expression(Wang et al. 2019a, b). Furthermore, TNF-α inhibits the proliferation of BMMSCs, promotes cellular senescence(Fan et al. 2022), and inhibits their ability to differentiate in multiple directions, thereby impairing stem cell functions(Wang et al. 2023; Qiu et al. 2019). Therefore, finding ways to reduce the functional impairment of BMMSCs by TNF-α is essential to improve the function of BMMSCs.

The study showed that AS attenuated the damaging effects of TNF-α on BMMSCs stemness, including the ability to promote cell proliferation, osteogenic and chondrogenic differentiation of BMMSCs. Previous studies have also demonstrated AS’s positive impact on BMMSCs functions, such as regulating stem cell proliferation, promoting osteogenic, chondrogenic, cardiogenic differentiation, and the immunomodulatory properties of BMMSCs(Hao et al. 2018; Cao et al. 2015; Zhan et al. 2018; Wang et al. 2023; Tang et al. 2014). The results indicate that AS may enhance the function of BMMSCs and improve the efficacy of cell transplantation.

YAP in the Hippo pathway is involved in the regulation of BMMSC stemness maintenance by AS. TNF-α was found to impaire the stemness of BMMSCs by down-regulating YAP, whereas AS treatment reversed this effect by up-regulating YAP. Furthermore, the results demonstrated that the protective effect of AS on the stemness of BMMSCs was partially reversed by YAP knockdown. Conversely, overexpression of YAP produced an effect similar to that of AS treatment, which provides compelling evidence that AS exerts its protective effect on the stemness of BMMSCs through the up-regulation of YAP. Previous studies have also shown that YAP regulates various functions of BMMSCs. Wang et al. (Wang et al. 2019a, b). found that YAP mediates the maintenance of BMMSC stemness by melatonin, while Chen et al. (Chen et al. 2020). found that YAP regulates brain damage after treatment of BMMSCs with cerebral haemorrhage in mice. Additionally, YAP is involved in the osteogenic and chondrogenic differentiation of BMMSCs and the regulation of apoptosis(Wang et al. 2020a, b, 2021a, b, 2023). These findings suggest that YAP may be a target molecule for regulating the function of BMMSCs.

This study demonstrated that YAP may regulate the stemness-maintaining effect of AS on BMMSC by modulating SMAD7 expression. SMAD7 expression was found to be downregulated after overexpression of YAP, and protein binding between YAP and SMAD7 was confirmed by Co-IP assay. SMAD7 overexpression after YAP overexpression partially reversed the protective effect of YAP overexpression on stemness damage in BMMSCs. Furthermore, the knockdown of SMAD7 in BMMSCs attenuated the damaging effects of TNF-α on BMMSC stemness. These findings indicate that SMAD7 is involved in regulating the stemness of BMMSCs. Similarly, previous studies showed that SMAD7 was involved in the functional regulation of BMMSCs. For example, Jiang et al. (Jiang et al. 2020). found that exosomes from BMMSCs promoted skin wound healing in rats by upregulating the expression of SMAD7. Similarly, Wang et al. (Wang et al. 2022). found that the possible mechanism of BMMSCs in the treatment of Peyronie’s disease was related to the increased expression of SMAD7. Additionally, SMAD7 plays a role in regulating the process of osteogenic and adipogenic differentiation in BMMSCs(Li et al. 2017; Wang et al. 2019a, b; Feng et al. 2022). Taken together, SMAD7 could be a potential molecular target for regulating BMMSC functions.

In this study, we used the STRING protein database to predict the potential binding between YAP and SMAD7. We further validated the binding between YAP and SMAD7 through co-immunoprecipitation (Co-IP) and chromatin immunoprecipitation (ChIP) experiments and found that overexpression of YAP inhibited the expression of SMAD7, suggesting a negative regulatory relationship between the two. Previous studies have also suggested a negative regulatory relationship between YAP and SMAD7, similar to the results of the present study. Studies have shown that SMAD7 interacts with YAP and can inhibit YAP expression by promoting YAP degradation (Li et al. 2024; Ma et al. 2019). In addition, Hong et al. found that miR-21-3p upregulated YAP expression by directly inhibiting SMAD7 (Hong et al. 2021). However, some studies have suggested a positive relationship between the two. Jiang et al. demonstrated that YAP inhibition in primary human nasal epithelial cells treated with a YAP inhibitor resulted in a decrease in Smad7 expression (Jiang et al. 2023). Wang et al. showed that SMAD7 promotes hepatocellular carcinoma by activating the YAP/NOTCH pathway (Wang et al. 2020a, b). These studies suggest that YAP and SMAD7 may regulate each other and be involved in the regulation of bodily functions.

This study found that AS delayed the stemness-damaging effect of TNF-α on BMMSCs. Mechanistically, AS may exert this reversal effect by upregulating the expression of YAP and downregulating the expression of SMAD7. The results of this study are expected to advance the application of AS to improve the efficacy of cell transplantation. However, this study has several limitations: (1) The mechanism by which AS affects the upregulation of YAP remains unclear; (2) The process by which YAP binds to SMAD7 and causes its down-regulation is unresolved; (3) This research lacks validation from in vivo study.

## Conclusions

This study found that AS can reverse the damaging effects of TNF-α on BMMSCs stemness. Mechanistically, AS may exert these effects by modulating the YAP-SMAD7 axis. The study provides an experimental basis to advance the application of AS in stem cell tissue engineering.

### Electronic supplementary material

Below is the link to the electronic supplementary material.

Supplementary Material 1

Supplementary Material 2

## Data Availability

The datasets supporting the conclusions of this article are included within the article.
