# Supplementary material for: Aspirin attenuates the detrimental effects of TNF-α on BMMSC stemness by modulating the YAP-SMAD7 axis
Source: Mol Med. 2024 Aug 16;30:126. doi: 10.1186/s10020-024-00890-z (PMC11330132; doi:10.1186/s10020-024-00890-z)
Supplement: Supplementary file 2 — Supplementary Material 2 [file 10020_2024_890_MOESM2_ESM.docx]

**Aspirin attenuates the detrimental effects of TNF-α on BMMSC stemness by modulating the YAP-SMAD7 axis**

**Supplementary Figures and figure legends**

**Supplementary Fig. 1**

**
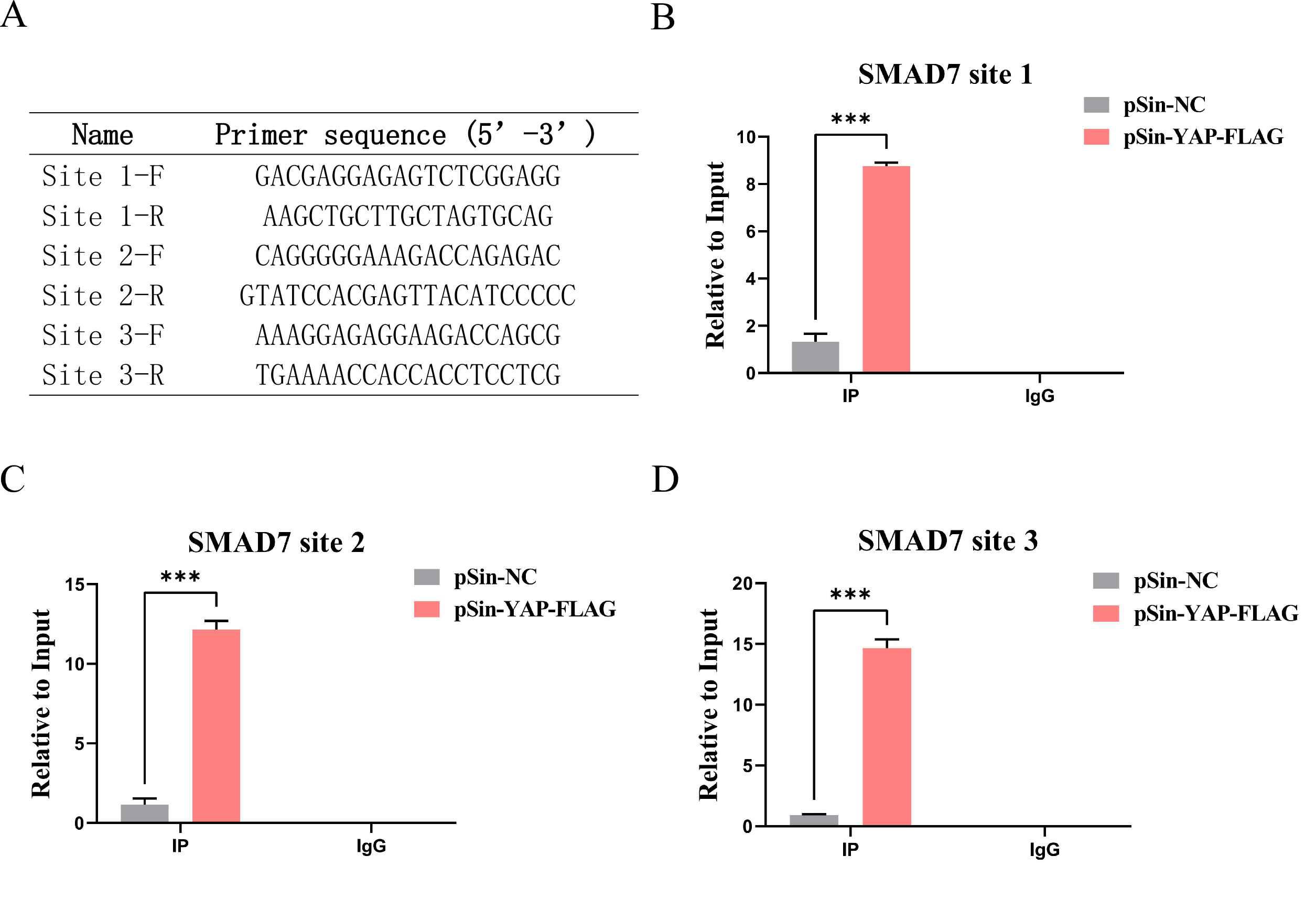
**

**Supplementary Fig.** **1** **Detection of YAP binding to SMAD7 using ChIP-qPCR assay.** (A) Sequences of primers used for ChIP-qPCR.; (B) Detection of YAP binding to SMAD7 site 1 using ChIP-qPCR assay; (C) Detection of YAP binding to SMAD7 site 2 using ChIP-qPCR assay; (D) Detection of YAP binding to SMAD7 site 3 using ChIP-qPCR assay; ****P*<0.001.
