# Supplementary material for: Aspirin attenuates the detrimental effects of TNF-α on BMMSC stemness by modulating the YAP-SMAD7 axis
Source: Mol Med. 2024 Aug 16;30:126. doi: 10.1186/s10020-024-00890-z (PMC11330132; doi:10.1186/s10020-024-00890-z)
Supplement: Supplementary file 1 — Supplementary Material 1 [file 10020_2024_890_MOESM1_ESM.doc]

**Dear Editors and Reviewers:**

Thank you for your constructive comments concerning our manuscript. "Aspirin attenuates the detrimental effects of TNF-α on BMMSC stemness by modulating the YAP-SMAD7 axis” (MOME-D-24-00548). We have considered the comments and suggestions carefully and made appropriate changes to our manuscript. We believe the alterations and additions have strengthened this manuscript and will meet with your approval. The revised sections are marked in the draft. Please see our responses to the specific comments below.

Reviewer #1:

In many respects, this paper presents novel functions of aspirin through YAP and SMAD7 to recover BMMSC stemness from TNF-α. However, there are several questions and suggestions that I believe will greatly strengthen the paper.

1. Please conduct quantitative analysis for all western blot results. It is important to show the statistical changes in each protein expression among the experimental groups.

Response: Thank you for your comments.We have supplemented the quantitative results of the western blot experiments in Fig 1-5 with your suggestions.

2. Please provide the GenBank accession number for each gene in Table 1.

Response: Thank you for your comments.We have supplemented the GenBank accession number for each gene in Table 1.

Table 1. Sequences of primers used for quantitative PCR.

| Gene | Genebank ID | Primer sequence (5’-3’) |
| --- | --- | --- |
| *GAPDH* | 2597 | Forward: GGAGCGAGATCCCTCCAAAAT;  Reverse: GGCTGTTGTCATACTTCTCATGG; |
| *C-MYC* | 4609 | Forward: GGCTCCTGGCAAAAGGTCA;  Reverse: CTGCGTAGTTGTGCTGATGT; |
| *NANOG* | 79923 | Forward: TTTGTGGGCCTGAAGAAAACT;  Reverse: AGGGCTGTCCTGAATAAGCAG; |
| *SOX2* | 6657 | Forward: GCCGAGTGGAAACTTTTGTCG;  Reverse: GGCAGCGTGTACTTATCCTTCT; |
| *OCN* | 632 | Forward: CACTCCTCGCCCTATTGGC;  Reverse: CCCTCCTGCTTGGACACAAAG; |
| *OPN* | 6696 | Forward: CTCCATTGACTCGAACGACTC;  Reverse: CAGGTCTGCGAAACTTCTTAGAT; |
| *RUNX2* | 860 | Forward: TGGTTACTGTCATGGCGGGTA;  Reverse: TCTCAGATCGTTGAACCTTGCTA; |
| *COL2A1* | 1280 | Forward: GCTGCGGATGCTCTCAATCT;  Reverse: TGGACGATCAGGCGAAACC; |
| *ACAN* | 176 | Forward: ACTCTGGGTTTTCGTGACTCT;  Reverse: ACACTCAGCGAGTTGTCATGG; |
| *SOX9* | 6662 | Forward: AGCGAACGCACATCAAGAC;  Reverse: CTGTAGGCGATCTGTTGGGG; |
| *YAP* | 10413 | Forward: TAGCCCTGCGTAGCCAGTTA;  Reverse: TCATGCTTAGTCCACTGTCTGT; |
| *SMAD7* | 4092 | Forward: TTCCTCCGCTGAAACAGGG;  Reverse: CCTCCCAGTATGCCACCAC; |

3. Please provide the Addgene plasmid number in the construction of plasmids part in the material section.

Response: Thank you for your suggestions. We have provided the Addgene plasmid number in the construction of plasmids part in the material section (Line 168-170).

Lentiviral transfer plasmids, pSin-YAP-FLAG(#66853) and pSin-SMAD7-FLAG(#141518), were created by cloning the human YAP and SMAD7 coding sequences into the plasmid pSin-puro.

4. Please provide each Yap and scramble shRNA sequence.

Response: Thank you for your suggestions. We have provided Yap and scramble shRNA sequence in the material section(Line 170-177).

To silence the expression of YAP and SMAD7, the shRNA sequence was cloned into the lentiviral transfer plasmids pSin-YAP shRNA(sequence: GCCACCAAGCTAGATAAAGAA) and pSin-SMAD7 shRNA(sequence: GCAGACAGACACTGGATTAAA). A control scramble-shRNA(sequence: GCGCGCTTTGTAGGATTCG) was also constructed.

5. Yap is co-transcriptional factor and usually regulates cell proliferation and/or differentiation through gene expression. Although the authors briefly mentioned this as a future study at the end of the discussion section, they should discuss more thoroughly how Yap regulates SMAD7. Are there references showing that Yap regulates SMAD7 expression directly or through upstream factor expression? A ChIP assay could clarify this part if necessary.

Response: Thank you for your valuable suggestions. In response to your input, we have added a discussion section to the article about the regulation of YAP and SMAD7 molecules (Line 339-357). However, there is currently no evidence to suggest whether YAP can regulate SMAD7 expression directly or indirectly through upstream molecular expression. Consequently, we have also conducted a ChIP-qPCR experiment (Supplementary Fig. 1) in accordance with your recommendation. The results demonstrate that YAP can regulate SMAD7 expression directly by binding to SMAD7.

In this study, we used the STRING protein database to predict the potential binding between YAP and SMAD7. We further validated the binding between YAP and SMAD7 through co-immunoprecipitation (Co-IP) and chromatin immunoprecipitation (ChIP) experiments and found that overexpression of YAP inhibited the expression of SMAD7, suggesting a negative regulatory relationship between the two. Previous studies have also suggested a negative regulatory relationship between YAP and SMAD7, similar to the results of the present study. Studies have shown that SMAD7 interacts with YAP and can inhibit YAP expression by promoting YAP degradation (29, 30). In addition, Hong et al. found that miR-21-3p upregulated YAP expression by directly inhibiting SMAD7 (31). However, some studies have suggested a positive relationship between the two. Jiang et al. demonstrated that YAP inhibition in primary human nasal epithelial cells treated with a YAP inhibitor resulted in a decrease in Smad7 expression (32). Wang et al. showed that SMAD7 promotes hepatocellular carcinoma by activating the YAP/NOTCH pathway (33). These studies suggest that YAP and SMAD7 may regulate each other and be involved in the regulation of bodily functions.

Supplementary Fig. 1

Supplementary Fig. 1 Detection of YAP binding to SMAD7 using ChIP-qPCR assay. (A) Sequences of primers used for ChIP-qPCR.; (B) Detection of YAP binding to SMAD7 site 1 using ChIP-qPCR assay; (C) Detection of YAP binding to SMAD7 site 2 using ChIP-qPCR assay; (D) Detection of YAP binding to SMAD7 site 3 using ChIP-qPCR assay; ***P<0.001.

6. Further, even though Yap is a major factor in the Hippo pathway, regulating cell proliferation in various cells, no research in this part has been included in this article. The authors need to explain why they did not consider Hippo pathway-related genes such as Tead4 and Lats1/2.

Response: We are grateful for your valuable suggestions. In this study, we chose to investigate the function of YAP molecule primarily based on the following two points: Firstly, our team's published articles in 2019 (1) indicated that YAP was involved in regulating the stemness maintenance of BMMSCs. This suggested that YAP in the Hippo pathway play an important role in stem cell stemness maintenance. Furthermore, the significant regulatory role of YAP molecule was also fully verified in our present study. Secondly, in the preliminary experiments conducted for this study, it was hypothesized that other molecules in the Hippo pathway, such as Tead4 and Lats1/2, might play a regulatory role in stem cell stemness maintenance. However, following treatment of BMMSCs with TNF-alpha and aspirin, it was observed that the expression of molecules such as Tead4 and Lats1/2 did not demonstrate significant changes, as determined by qRT-PCR and western blot, indicating that these molecules may not be involved in the regulation of stemness maintenance in stem cells. Consequently, subsequent studies were conducted with a focus on the YAP rather than other molecules of the Hippo pathway.

References：

(1) Wang Xudong, Liang Tongzhou, Qiu Jincheng et al. Melatonin Reverses the Loss of Stemness Induced by TNF-α in Human Bone Marrow Mesenchymal Stem Cells through Upregulation of YAP Expression.[J].Stem Cells Int, 2019, 2019: 6568394. doi: 10.1155/2019/6568394.

7. Similarly, in Fig4A, the authors showed the STRING result, indicating that Yap has the potential to bind to SMAD7, TP73, etc. However, because the interaction between Yap and Smad7 is well known and the authors did not investigate other factors, I feel this result is not so critical to this study. They should explain more clearly why they chose SMAD7 over other Hippo factors.

Response: Thank you for your valuable suggestions. The protein interactions predicted by the STRING website suggest that YAP may associate with molecules such as SMAD7, TP73 and AMOT. In this article, the pre-experiment first explored the binding relationship between YAP and SMAD7 or TP73, but the results of the Co-IP experiment suggested that YAP binds to SMAD7 but not to TP73 and other molecules, and the ChIP experiment further confirmed that YAP binds to SMAD7. In addition, there is sufficient evidence in the literature that the Hippo pathway cross-talks with the TGF-β pathway. Therefore, we focused our study on the SMAD7 rather than other molecules.

8. In Fig4E, please provide quantitative result of colony formation as done for other figures.

Response: Thank you for your suggestions. We have provided quantitative result of colony formation in Fig 4G.

Reviewer #2:

In this manuscript, by Wang et al., the authors analysed the effects of Aspirin (AS) and TNF-α on BMMSC stemness and molecular mechanisms.

They found that AS treatment attenuates TNF-α-induced damage on BMMSC stemness through upregulation of YAP and inhibition of SMAD7 expression.

The results suggest how AS can be used to improve the efficacy of cell transplantation and how this study provides an experimental basis for advancing the application of AS in tissue engineering of stem cells.

My main concerns are the following:

1. In the introduction from line 79 to 86, I suggest summarising and possibly eliminate the methods, which are in any case present in the materials and methods, and focus better on the aim of the study.

Response: Thank you for your valuable suggestions. We have made changes to the manuscript based on your suggestions(Line 79-81).

In this study, we aim to explore the roles and molecular mechanisms of AS and TNF-α on BMMSC stemness, which may advance the application of AS to improve the efficacy of stem cell therapy.

2. In the Materials and Method, please reported Ethical approved and the reference.

Response: We are grateful for your valuable suggestions. We have add Ethical approved and the reference(Line 85-90, Line 177-178).

This study has been approved by the the Research Ethics Committee of the First Affiliated Hospital of Sun Yat-sen University. Written informed consent was obtained from each volunteer. Referring to previously published literature(8, 12, 14-17), the BMMSCs used in this study were extracted from healthy volunteers and isolated using density gradient centrifugation (500 g for 20 min at 37℃).

Lentiviral infection was performed according to the procedure outlined in a previously published article(18).

3. In the Materials and Method paragraph 1 (from line 89 to 96), please enter the speed, time and temperature of the density gradient centrifuge used as well as the concentrations of the different medium supplemented used.

Response: Thank you for your valuable suggestions. We have made changes to the manuscript based on your suggestions(Line 88-92).

the BMMSCs used in this study were extracted from healthy volunteers and isolated using density gradient centrifugation (500 g for 20 min at 37℃). They were then cultured in a low-sugar DMEM medium supplemented with 10% fetal bovine serum (FBS), penicillin(100 U/ml), and streptomycin(100 U/ml).

4. In the Materials and Method paragraph 2 (from line 98 to 104), please enter all the code of the specific antibody that you reported.

Response: Thank you for your valuable suggestions. We have made changes to the manuscript based on your suggestions(Line 99-105).

Antibodies against C-MYC(ab32072), NANOG(ab109250), SOX2(ab92494), OCN(ab133612), OPN(ab214050), RUNX2(ab192256), COL2A1(ab34712), ACAN(ab3778), SOX9(ab185966), YAP(ab52771), SMAD7(ab216428) and GAPDH(ab9485) were purchased from Abcam (Cambridge, UK). Goat anti-mouse IgG H&L (HRP)(#14709) and Goat anti-rabbit IgG H&L (HRP)(#35401) were purchased from Cell Signaling Technology (Boston, USA).

5. In the Materials and Method paragraph 6, do all primary antibodies have the same dilution? Which chemiluminescence product did you use? Please, report it.

Answer: We are grateful for your valuable suggestions. Yes, all primary antibodies have the same dilution 1:1000. Besides, we have added chemiluminescence product in the manuscript (Line 150-152).

The protein bands were then visualized using an HRP Chemiluminescent Western Blot Detection Kit (Merck Millipore, USA).

6. Report in all 5 figures the graphs of the protein quantifications associated with the respective western blots.

Response: Thank you for your comments.We have supplemented the quantitative results of the western blot experiments in Fig 1-5 with your suggestions.

7. In the discussion part, I would remove some of the bibliography and leave a little more space for the discussion of your results, highlighting more concretely what the future prospects may be.

Response: Thank you for your suggestions. We have made changes to the manuscript based on your suggestions(Line 264-277, Line 306-311, Line 339-357).

Given the paucity of research exploring the impact of AS on the maintenance of stem cell stemness, this study makes a novel contribution to this field by demonstrating that AS mitigates damage of the inflammatory factor TNF-α on the stemness of BMMSCs. Mechanistically, AS may exert this reversal effect by upregulating the expression of YAP and downregulating the expression of SMAD7. The innovations of this study include the following two main points: 1. The first to propose that AS can delay inflammation-induced stemness damage of BMMSCs; 2. The first to propose the important regulatory role of the YAP-SAMD7 signalling axis in maintaining the stemness of BMMSCs. The results of this study are expected to promote the application of AS in maintaining the stemness of BMMSCs, thereby improving the efficacy of cell transplantation and providing new hope for the treatment of many difficult and complicated diseases.

Furthermore, the results demonstrated that the protective effect of AS on the stemness of BMMSCs was partially reversed by YAP knockdown. Conversely, overexpression of YAP produced an effect similar to that of AS treatment, which provides compelling evidence that AS exerts its protective effect on the stemness of BMMSCs through the up-regulation of YAP.

In this study, we used the STRING protein database to predict the potential binding between YAP and SMAD7. We further validated the binding between YAP and SMAD7 through co-immunoprecipitation (Co-IP) and chromatin immunoprecipitation (ChIP) experiments and found that overexpression of YAP inhibited the expression of SMAD7, suggesting a negative regulatory relationship between the two. Previous studies have also suggested a negative regulatory relationship between YAP and SMAD7, similar to the results of the present study. Studies have shown that SMAD7 interacts with YAP and can inhibit YAP expression by promoting YAP degradation (29, 30). In addition, Hong et al. found that miR-21-3p upregulated YAP expression by directly inhibiting SMAD7 (31). However, some studies have suggested a positive relationship between the two. Jiang et al. demonstrated that YAP inhibition in primary human nasal epithelial cells treated with a YAP inhibitor resulted in a decrease in Smad7 expression (32). Wang et al. showed that SMAD7 promotes hepatocellular carcinoma by activating the YAP/NOTCH pathway (33). These studies suggest that YAP and SMAD7 may regulate each other and be involved in the regulation of bodily functions.
